# Supplementary material for: Can syndromic surveillance help forecast winter hospital bed pressures in England?
Source: PLoS One. 2020 Feb 10;15(2):e0228804. doi: 10.1371/journal.pone.0228804 (PMC7010388; doi:10.1371/journal.pone.0228804)
Supplement: S1 Table — (DOCX) [file pone.0228804.s001.docx]

***Table S1: Absolute mean forecast errors (daily admissions) in England by age band, stratified by season.***

| **Syndromic indicator in model**  **Season** | **Age band (years)** | | | | | | |
| --- | --- | --- | --- | --- | --- | --- | --- |
|  | **under 1** | **1 to 4** | **5 to 14** | **15 to 44** | **45 to 64** | **65 to 74** | **over 75** |
| **Null model** |  |  |  |  |  |  |  |
| 2013/2014 | 24.44 | 26.71 | 18.69 | 47.68 | 37.24 | 25.92 | 51.54 |
| 2014/2015 | 23.34 | 27.94 | 19.23 | 46.66 | 34.80 | 27.41 | 58.61 |
| 2015/2016 | 23.58 | 27.33 | 20.44 | 42.78 | 33.66 | 24.99 | 48.95 |
| 2016/2017 | 22.83 | 25.71 | 20.01 | 39.97 | 34.75 | 26.33 | 57.24 |
| 2017/2018 | 27.61 | 32.18 | 21.60 | 49.91 | 39.94 | 30.31 | 60.42 |
| **EDSSS: ARI** |  |  |  |  |  |  |  |
| 2013/2014 | 18.48 | 25.60 | 18.72 | 46.54 | 37.39 | 25.91 | 50.31 |
| 2014/2015 | 17.82 | 23.59 | 19.20 | 45.34 | 36.40 | 26.87 | 54.20 |
| 2015/2016 | 20.65 | 26.12 | 20.45 | 42.86 | 33.59 | 25.19 | 49.81 |
| 2016/2017 | 20.53 | 22.56 | 20.04 | 40.68 | 34.97 | 26.51 | 54.86 |
| 2017/2018 | 20.58 | 34.82 | 20.01 | 46.74 | 37.20 | 27.66 | 55.87 |
| **EDSSS: admitted** |  |  |  |  |  |  |  |
| 2013/2014 | 22.33 | 25.51 | 18.82 | 47.24 | 37.81 | 26.15 | 50.78 |
| 2014/2015 | 20.17 | 25.51 | 19.26 | 46.70 | 35.06 | 27.41 | 58.61 |
| 2015/2016 | 20.80 | 26.03 | 20.63 | 43.62 | 33.66 | 25.01 | 49.16 |
| 2016/2017 | 21.33 | 25.60 | 19.77 | 40.66 | 35.07 | 26.55 | 58.98 |
| 2017/2018 | 25.18 | 33.97 | 20.24 | 49.02 | 40.82 | 32.37 | 88.68 |
| **EDSSS: bronchitis** |  |  |  |  |  |  |  |
| 2013/2014 | 17.72 | 25.55 | 18.72 | 47.67 | 37.28 | 26.09 | 52.04 |
| 2014/2015 | 17.46 | 26.12 | 19.27 | 46.92 | 34.97 | 27.28 | 57.87 |
| 2015/2016 | 19.57 | 27.68 | 20.43 | 42.85 | 33.59 | 25.00 | 48.40 |
| 2016/2017 | 20.50 | 26.68 | 20.16 | 40.25 | 34.57 | 26.38 | 56.76 |
| 2017/2018 | 19.09 | 34.22 | 19.92 | 46.97 | 36.83 | 28.31 | 57.03 |
| **EDSSS: pneumonia** |  |  |  |  |  |  |  |
| 2013/2014 | 23.34 | 26.12 | 18.69 | 47.36 | 37.62 | 26.33 | 50.09 |
| 2014/2015 | 23.37 | 27.53 | 19.51 | 46.78 | 35.13 | 27.28 | 56.52 |
| 2015/2016 | 23.11 | 27.21 | 20.58 | 43.63 | 33.59 | 25.05 | 51.65 |
| 2016/2017 | 22.72 | 24.86 | 19.99 | 40.09 | 34.72 | 26.17 | 54.34 |
| 2017/2018 | 24.16 | 30.73 | 19.78 | 46.27 | 36.90 | 28.52 | 59.45 |
| **GPIHSS: LRTI** |  |  |  |  |  |  |  |
| 2013/2014 | 13.88 | 20.20 | 19.23 | 46.71 | 37.90 | 26.42 | 42.94 |
| 2014/2015 | 13.54 | 19.70 | 19.64 | 46.18 | 36.66 | 26.74 | 47.12 |
| 2015/2016 | 15.80 | 23.48 | 19.94 | 44.87 | 33.39 | 24.14 | 44.67 |
| 2016/2017 | 13.54 | 21.75 | 19.80 | 39.66 | 34.18 | 24.92 | 47.97 |
| 2017/2018 | 14.29 | 24.95 | 20.86 | 49.15 | 38.42 | 27.47 | 54.02 |
| **GPIHSS: ILI** |  |  |  |  |  |  |  |
| 2013/2014 | 23.58 | 26.53 | 18.56 | 47.31 | 37.62 | 26.33 | 49.56 |
| 2014/2015 | 22.94 | 27.49 | 18.95 | 46.55 | 35.01 | 27.21 | 54.49 |
| 2015/2016 | 23.14 | 26.00 | 19.49 | 43.15 | 33.76 | 24.93 | 46.17 |
| 2016/2017 | 22.74 | 25.30 | 19.98 | 40.47 | 34.60 | 25.66 | 50.91 |
| 2017/2018 | 28.62 | 38.13 | 21.46 | 53.53 | 42.19 | 31.86 | 85.72 |
| **GPIHSS: pneumonia** |  |  |  |  |  |  |  |
| 2013/2014 | 23.66 | 24.41 | 18.76 | 47.58 | 37.79 | 26.90 | 56.78 |
| 2014/2015 | 22.77 | 26.27 | 19.20 | 46.74 | 35.10 | 27.82 | 55.75 |
| 2015/2016 | 23.11 | 26.03 | 20.56 | 44.52 | 33.84 | 24.95 | 47.40 |
| 2016/2017 | 22.05 | 25.54 | 20.00 | 39.99 | 34.75 | 26.18 | 54.29 |
| 2017/2018 | 28.69 | 31.30 | 21.17 | 49.53 | 39.32 | 29.58 | 57.41 |
| **GPIHSS: URTI** |  |  |  |  |  |  |  |
| 2013/2014 | 16.00 | 17.93 | 17.86 | 46.65 | 37.90 | 26.52 | 42.58 |
| 2014/2015 | 15.61 | 19.70 | 19.78 | 46.42 | 36.46 | 26.92 | 47.06 |
| 2015/2016 | 17.24 | 18.83 | 18.08 | 44.36 | 33.56 | 24.10 | 44.90 |
| 2016/2017 | 17.61 | 19.47 | 18.99 | 40.16 | 34.18 | 24.66 | 47.27 |
| 2017/2018 | 17.07 | 23.54 | 19.21 | 49.56 | 38.65 | 27.44 | 54.09 |
| **GPOOHSS: bronchitis** |  |  |  |  |  |  |  |
| 2013/2014 | 17.03 | 24.02 | 18.81 | 47.84 | 37.42 | 26.13 | 52.05 |
| 2014/2015 | 15.95 | 25.96 | 19.10 | 46.63 | 34.82 | 27.00 | 57.51 |
| 2015/2016 | 18.59 | 26.81 | 20.37 | 43.58 | 33.82 | 25.34 | 48.12 |
| 2016/2017 | 16.56 | 26.11 | 20.35 | 40.15 | 34.83 | 26.06 | 57.23 |
| 2017/2018 | 17.38 | 29.47 | 21.02 | 48.90 | 39.26 | 29.38 | 58.23 |
| **GPOOHSS: ARI** |  |  |  |  |  |  |  |
| 2013/2014 | 17.03 | 21.59 | 18.76 | 45.88 | 37.83 | 26.57 | 46.82 |
| 2014/2015 | 17.90 | 22.69 | 19.38 | 45.21 | 36.25 | 26.54 | 48.64 |
| 2015/2016 | 18.71 | 24.88 | 20.55 | 45.54 | 33.44 | 24.76 | 46.65 |
| 2016/2017 | 18.48 | 23.01 | 19.97 | 41.35 | 34.32 | 24.77 | 47.92 |
| 2017/2018 | 17.24 | 27.57 | 21.13 | 49.66 | 38.66 | 27.75 | 57.09 |
| **NHS 111: cold/flu** |  |  |  |  |  |  |  |
| 2013/2014 | 21.70 | 22.73 | 16.73 | 43.18 | 34.37 | 23.66 | 43.94 |
| 2014/2015 | 23.92 | 28.72 | 19.33 | 46.66 | 35.22 | 26.98 | 52.76 |
| 2015/2016 | 23.69 | 25.48 | 20.23 | 43.18 | 33.89 | 24.95 | 46.59 |
| 2016/2017 | 22.67 | 24.87 | 20.09 | 40.26 | 34.24 | 25.58 | 48.26 |
| 2017/2018 | 26.61 | 35.40 | 20.62 | 59.10 | 41.15 | 30.91 | 74.48 |

EDSSS: emergency department syndromic surveillance system; GPIHSS: GP in-hours syndromic surveillance system; GPOOHSS: GP out of hours and unscheduled care syndromic surveillance system; NHS 111: telehealth syndromic surveillance system; ARI: acute respiratory infection; LRTI: lower respiratory tract infection; ILI: influenza-like illness; URTI: upper respiratory tract infection
